# Unexpected erythema nodosum during dupilumab treatment for severe asthma: Causal or casual?

**DOI:** 10.1016/j.jdcr.2026.03.059

**Published:** 2026-04-08

**Authors:** Federico Cusumano, Valeria Brazzelli, Ivan Taietti, Alessia Marseglia, Riccardo Castagnoli, Gian Luigi Marseglia, Amelia Licari

**Affiliations:** a Pediatric Unit, Department of Clinical, Surgical, Diagnostic, and Pediatric Sciences, University of Pavia, Pavia, Italy; b Pediatric Clinic, Fondazione IRCCS Policlinico San Matteo, Pavia, Italy; c Department of Clinical, Surgical, Diagnostic, and Pediatric Sciences, Institute of Dermatology, University of Pavia, Pavia, Italy; d Institute of Dermatology, Fondazione IRCCS Policlinico San Matteo, Pavia, Italy

**Keywords:** adverse drug reaction, biological treatment, dupilumab, erythema nodosum (EN), severe asthma

## Introduction

Erythema nodosum (EN) is the most common form of panniculitis in children. It is characterized by the sudden onset of painful, erythematous, subcutaneous nodules, primarily located in the pretibial areas. The cause of most EN cases is unknown, with idiopathic cases accounting for 23% up to 55% of occurrences.^1^^,^^2^ EN can also be secondary to various conditions, including infections, sarcoidosis, inflammatory bowel disease, medications, vaccinations, autoimmune disorders, and pregnancy.^3^ Generally, EN is a self-limiting disease, and most cases do not require treatment. In this report, we present the clinical case of a young woman with severe asthma who developed EN 1 year after starting treatment with dupilumab.

## Case report

**Fig 1:**
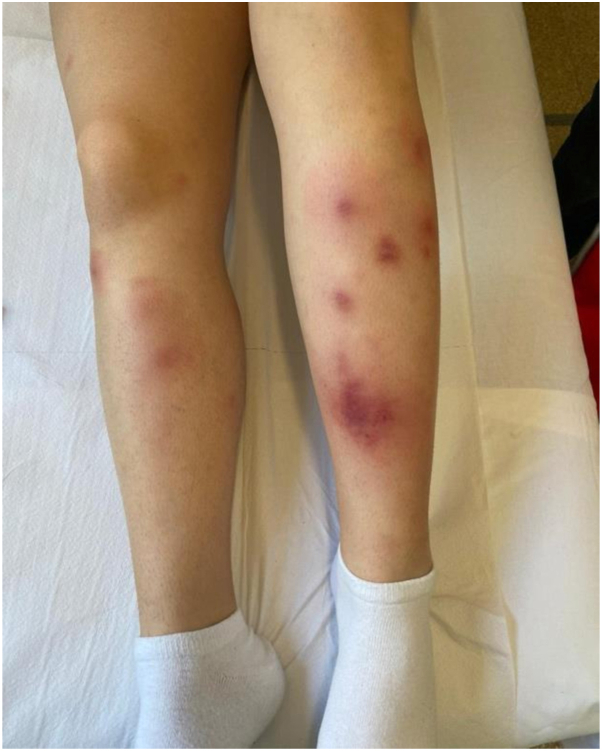
Clinical presentation of painful, erythematous, subcutaneous nodules on the pretibial regions in the reported patient.

**Fig 2:**
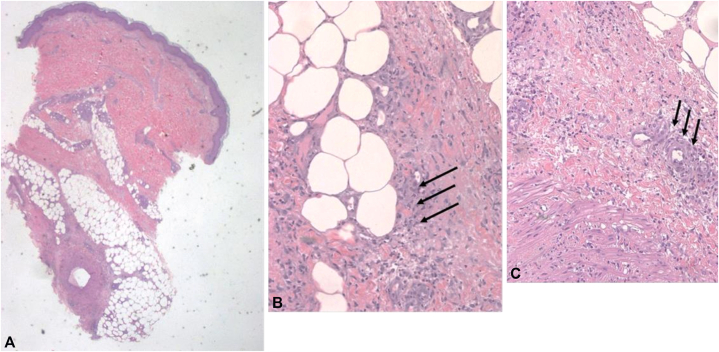
Histopathological findings show **(A)** a mostly septal panniculitis with thickened connective tissue septa. Superficial and deep perivascular inflammatory infiltrates, predominantly composed of lymphocytes and neutrophils, are also seen in the overlying dermis and between the more superficial fat lobules [hematoxylin–eosin stain, original magnification ×10]. **B,** Higher magnification demonstrates inflammatory infiltrates characterized by neutrophils, lymphocytes, and rare histiocytes, interstitially arranged between thick collagen bundles of the septa. The inflammatory infiltrates extend to the periphery of the adjacent fat lobules (*arrows*) [hematoxylin–eosin stain, original magnification ×40]. **C,** The inflammatory infiltrate is evident around vessels without vasculitis (*arrows*) [hematoxylin–eosin stain, original magnification ×40].

A 16-year-old woman is being followed by our Pediatric Allergology Unit for severe allergic asthma, rhinoconjunctivitis and sensitive to multiple allergens, including grasses, dust mites, birch, hazel, ragweed, mugwort, and pet dander from dogs and cats. Due to poorly controlled asthma while on fluticasone/formoterol and montelukast, she began treatment with dupilumab at the age of 15, achieving a good control of respiratory symptoms. After almost 1 year of treatment (34th dose), the patient developed bilateral erythematous painful nodules on the extensor surface of her lower legs (Fig 1). The skin lesions were not associated with systemic symptoms (fever, asthenia, weight loss), lymphadenopathy, cough, dyspnea, gastrointestinal symptoms (diarrhea, abdominal pain) or arthralgia. There were no recent reports of infections, fever, trauma, recent antibiotics and/or vaccines administrations, or any other drugs regular administration (eg, oral contraceptive pills). Her medical history included *Helicobacter pylori*-positive duodenitis, but there were no records of autoimmune or allergic diseases in her family medical history. A dermatological consultation raised suspicion for EN. This was therefore followed by a diagnostic workup that included blood tests, infection screenings, and imaging studies to rule infectious, autoimmune, and oncological triggering factors (Table I) Given fecal calprotectin levels at the upper limit of normal, along with complete absence of gastrointestinal symptoms, abdominal ultrasound and gastroenterologist consultation were performed, both of which were negative for inflammatory bowel disease. Since the results were negative, a histological biopsy was performed 1 month after symptom onset. The histological findings revealed a predominantly septal panniculitis, characterized by thickened connective tissue septa. Superficial and deep perivascular inflammatory infiltrates, predominantly lymphocytes, neutrophils, and rare histiocytes, interstitially arranged between thick collagen bundles, were seen in the overlying dermis and between the more superficial fat lobules, confirming the diagnosis of EN (Fig 2). After almost 6 weeks of persisting EN, treatment with dupilumab was discontinued (overall, after the 34th dose). Ibuprofen (400 mg twice daily) was initiated, followed by 1 week of betamethasone at a dose of 0.5 mg, subsequently tapered over 1 month, along with an 8-week topical therapy of clobetasol. During treatment, the lesions gradually regressed and healed completely after 8 weeks. Given the results of the blood, microbiological, and radiological tests, the positive clinical outcome after dupilumab was suspended, a diagnosis of iatrogenic EN was suspected by exclusion. Considering the good control of respiratory symptoms and the potential for symptom recurrence, dupilumab was therefore definitively suspended and treatment with fluticasone/formoterol and montelukast alone was continued. Follow-ups were continued in our Unit and no more EN exacerbations have been reported for more than 2 years.

**Table I:** Diagnostic assessment performed in the presented patient

Complete blood count		Infectious screening	
Hemoglobin	13.6 g/dL (n.v. 11.7-15.5)	ASLO titer	123.5 IU/ml (0.0-400.0 IU)
RBC	4.49 × 10^6^/uL (n.v. 3.80-5.20)	Oropharyngeal swab	Negative for bacteria and fungi
Hematocrit	40.6% (n.v. 35.0-45.0)	Serologies for EBV	
		IgG VCA	<20 U/ml (>20 positive)
		IgM VCA	<20 U/ml (>20 positive)
		IgG EBNA	<5 U/ml (>20 positive)
WBC	6.49 × 10^3^/uL (n.v. 4.00-10.00)	Serologies for CMV	
Neutrophils	5.01 × 10^3^/uL (n.v. 2.0-8.0)	IgM VCA	<18 U/ml (>22 positive)
Lymphocytes	0.83 × 10^3^/uL (n.v. 1.5-4.0)	IgG VCA	IgG <12 U/ml (>14 positive)
Monocytes	0.53 × 10^3^/uL (n.v. 0.1-1.0)	Serologies for *Mycoplasma pneumoniae*	
Eosinophils	0.11 × 10^3^/uL (n.v. 0.1-0.5)	IgG	<0.1 AU/ml (>10 positive)
Basophils	0.01 × 10^3^/uL (n.v. 0.00-0.2)	IgM	2.4 AU/ml (>10 positive)
Platelets	206 × 10^3^/uL (n.v. 150-450)	Serologies for HBV	
Inflammatory markers		HBsAg	Negative
CRP	0.05 mg/dL (n.v. < 0.5)	Anti-HBsAg	<10 mUI/mL (>10, immunity present)
ESR	15.0 mm/h (n.v < 15)	Anti-HBc	Negative
Faecal calprotectin	157.0 ng/mg (n.v. 0.0-50.0)	Serologies for HCV	Anti-HCV negative
Coagulation profile		Stool cultures for *Campylobacter*, *Yersinia*, *Salmonella, Shigella*	Negative
		*Helicobacter pylori* antigen stool test	Negative
Prothrombinemia	101.00% (n.v. 70.00-120.00)	Tubercolosis screening	
INR	0.99 (n.v. 0.90-1.20)	QuantiFERON-TB	Negative
aPTT	21.00 s (n.v. 20.00-32.00)	Tuberculin skin test	Negative
PTT - ratio	0.74 (n.v. 0.70-1.12)	Autoimmune screening	
Antithrombin III	105.60% (n.v. 70.00-120.00)		
Fibrinogen	344 mg/dL (n.v. 170.00-410.00)	ANA	Positive (1:80)
Protein C	105% (n.v. 70-140)	Anti-ENA screening	Nuclear fine speckled negative
Protein S	85.00% (n.v. 60.00-130.00)	Anti-dsDNA	0.8 UI/ml (<10, negative)
Metabolic and hormonal panel		ASCA	
Creatinine	0.75 mg/dL (n.v. 0.55-1.02)	IgA	1.0 U/ml (n.v. < 7)
		IgG	0.7 U/ml (n.v. < 7)
Urea	24.0 mg/dL (n.v. 10.0-50.0)	ANCA	
GOT	18 UI/L (n.v. 11.0-39.0)	Anti-MPO	0.0 U/mL (n.v. < 2)
		Anti PR3	0.0 U/mL (n.v. < 3.5)
GPT	18 UI/L (n.v. 11.0-34.0)	AMA	Negative (n.v. < 1:40)
GGT	17.0 U/L (n.v. 11.0-53.0)	APCA	Negative (n.v. < 1:40)
Total bilirubin	0.47 mg/dL (n.v. 0.2-1.1)	Anti-TPO	<10.0 IU/mL (n.v. < 40)
Glycemia	84.0 mg/dL (n.v. 76.0-100.0)	Anti-TG	<20.0 IU/mL (n.v. < 20)
		Anti-TSHr	<10.0 IU/L (n.v. < 0.55)
Total cholesterol	160 mg/dL (n.v. < 200)	ATTGA	0.2 U/ml (n.v. < 7)
Tryglicerides	107 mg/dL (n.v. 40.0-160.0)	ACE	32.0 U/L (n. v. 13.3-63.9)
Amylase	64.0 mU/ml (n.v. 25.0-125.0)	Imaging studies	
Lypase	37.0 mU/ml (n.v. 8.0-58.0)	Chest X-ray	No pathological findings
TSH	0.922 mIU/L (n.v. 0.400-4.000)	Abdominal ultrasound	No pathological findings
LH	0.5 IU/L (n.v.: follicular phase 1.1-11.6; periovulatory phase 17-77, luteinic phase <14.7)	Histopathological examination	
FSH	0.1 IU/L (n.v.: follicular phase 2.8-11.3, periovulatory phase 5.8-21.0, luteinic phase 1.2-9.0)	Histological examination	Erythema nodosum pattern
Androstenedione	3.60 mg/mL (n.v. 0.46-3.39)	Immunofluorescence assay	Negative

*ACE*, Angiotensin converting enzyme; *AMA*, anti-mitochondrial antibodies; *ANA*, anti-nuclear antibodies; *ANCA*, anti-neutrophil cytoplasmic antibodies; *anti-dsDNA*, anti-double stranded DNA; *APCA*, anti-parietal cell antibodies; *aPTT*, activated partial thromboplastin time; *ASCA*, anti-Saccharomyces cerevisiae antibodies; *ASLO*, anti-streptolysin-O; *ATTGA*, anti-tissue transglutaminase antibodies; *CRP*, C-reactive protein; *EBN*, Epstein-Barr nuclear antigen; *EBV*, Ebstein-Barr virus; *ENA*, extractable nuclear antigen; *ESR*, erythrocyte sedimentation rate; *FSH*, follicle stimulating hormone; *GGT*, gamma-glutamyl transferase; *GOT*, glutamic oxaloacetic transaminase; *GPT*, glutamate-pyruvate transaminase; *HBc*, hepatis B core protein; *HBsAg*, hepatitis B surface antigen; *HBV*, hepatitis B virus; *HCV*, hepatitis C virus; *INR*, international normalized ratio; *IU*, international unit; *LH*, luteinizing hormone; *MPO*, myeloperoxidase; *PR3*, proteinase-3; *RBC*, red blood cell; *TG*, thyroglobulin; *TPO*, thyroid peroxidase; *TSH*, thyroid-stimulating hormone; *TSHr*, thyrotropin receptor; *VCA*, viral capsid antigen; *WBC*, white blood cell.

## Discussion

EN is relatively uncommon in pediatric patients, yet it is recognized as the most prevalent form of panniculitis in this age group. While many cases of EN are deemed idiopathic, secondary forms are often linked to infectious agents. Abu-Rumeileh et al reported an etiological diagnosis in more than half of patients in their cohort of 68 pediatric patients with EN: infection-related EN were the most frequently reported (42.6%), followed by Crohn’s disease (8.8%), celiac disease (1.5%), Sjogren syndrome (1.5%), and Hodgkin lymphoma (1.5%). Idiopathic EN was reported in 44.1% of the patients.^4^ The precise pathogenesis of EN remains unclear. Some researchers consider EN as a delayed-type hypersensitivity reaction triggered by exposure to various internal or external antigens. This reaction is thought to involve the deposition of immune complexes in the septal venules of the subcutaneous fat.^5^ The main drugs known to trigger EN include penicillin, macrolides, and cephalosporins.^5^ Dupilumab is known for its safety profile. Its most common adverse effects observed in patients with asthma are an increased eosinophil count, injection site reactions, and a rise in upper respiratory tract infections. Only 3 cases of EN in adult patients suffering from severe asthma while undergoing dupilumab treatment have been reported in literature (Table II).^6^, ^7^, ^8^ EN typically appears after the first administrations of the drug, with a latency period of approximately 7 weeks. Laboratory findings in 2 of the cases indicated a significant increase in the eosinophil counts, which is another adverse effect linked to dupilumab.^6^^,^^7^ In the first case reported by Hanami et al, a skin biopsy revealed lobular panniculitis characterized by a predominantly mononuclear infiltrate.^6^ Conversely, the case documented by Mustin et al showed septal panniculitis, which exhibited a lymphohistiocytic and granulomatous infiltrate containing scattered eosinophilic elements.^7^ Importantly, neither biopsy displayed any vascular findings.^6^^,^^7^ Following the diagnosis, dupilumab was discontinued in all 3 cases, and treatment with steroids or anti-inflammatory drugs was initiated, resulting in complete resolution of the symptoms. In the cases reported by Mustin et al and Mroczka et al, a second cycle of dupilumab was attempted; however, this led to an early recurrence of EN.^7^^,^^8^ To our knowledge, EN has been only rarely reported during treatment with dupilumab in pediatric patients. In this case, EN had a significantly longer latency period than in adult patients and required a combination of steroid and NSAID therapy for an extended period to achieve complete remission. Due to this adverse event and the possible recurrence of symptoms following re-exposure to the drug as highlighted by a case reported in literature,^6^ dupilumab therapy was permanently discontinued. However, the patient continued regular treatment with fluticasone/formoterol and montelukast, without any worsening of their asthma condition. Medical history was negative for recent intercurrent infections and drug therapies leading to EN, in particular, antibiotics, vaccines and oral contraceptive therapy. A skin biopsy confirmed the diagnosis of EN after blood, microbiological and radiological investigations ruled out the main causes, indicating a likely potential iatrogenic etiology associated with dupilumab. While these findings raise the possibility of a drug-related etiology, they do not allow a definitive conclusion regarding causality. Notably, no other laboratory findings typically associated with dupilumab therapy, such as eosinophilia, were observed. EN during dupilumab therapy is a rare adverse event and may necessitate reconsidering the continuation of treatment.^9^ Therefore, it is important to confirm the diagnosis through a skin biopsy of the lesion and rule out any underlying secondary conditions. Careful clinical monitoring, including a dermatological assessment, is essential to evaluate the treatment of the lesions and monitor their progression.

**Table II:** Comparison of the main features of temporal association between erythema nodosum and dupilumab reported in the literature

Case report	Age	Sex	Ethnicity	Allergic disease	EN onset	Lab test	Biopsy	Treatment
Hanami et al^6^	49	Male	Asian	Asthma, AD	2nd dose	Elevated CRP	Yes	NSAID
Mroczka et al^8^	52	Female	Caucasian	Asthma	6th dose	Eosinophilia	No	Systemic steroids
Mustin et al^7^	74	Female	Caucasian	Asthma, CRSwNP	5th dose	Eosinophilia	Yes	NSAID + systemic steroids
Our patient	16	Female	Caucasian	Asthma, rhinocongiuntivits	34th dose	Normal	Yes	NSAID + systemic and topical steroids

*AD*, Atopic dermatitis; *CRSwNP*, chronic rhinosinusitis with nasal poliposis; *EN*, erythema nodosum; *NSAID*, non-steroidal anti-inflammatory drugs.

## Conflicts of interest

None disclosed.
